# Relationships between Training Loads and Selected Blood Parameters in Professional Soccer Players during a 12-Day Sports Camp

**DOI:** 10.3390/ijerph17228580

**Published:** 2020-11-19

**Authors:** Łukasz Radzimiński, Zbigniew Jastrzębski, Guillermo F. López-Sánchez, Andrzej Szwarc, Henryk Duda, Aleksander Stuła, Jacek Paszulewicz, Paul Dragos

**Affiliations:** 1 Department of Physiology, Gdansk University of Physical Education and Sport, K. Górskiego 1, 80-336 Gdansk, Poland; zb.jastrzebski@op.pl; 2 Faculty of Sport Sciences, University of Murcia, 30720 Murcia, Spain; gfls@um.es; 3 Department of Sport Sciences, Gdansk University of Physical Education and Sport, K. Górskiego 1, 80-336 Gdansk, Poland; andrzej.szwarc@awf.gda.pl; 4 Department of Sport Sciences, Krakow University of Physical Education, 31-571 Kraków, Poland; hendud@op.pl; 5 Department of Physical Education and Physiotherapy, Opole University of Technology, 45-758 Opole, Poland; awfstula@poczta.onet.pl; 6 Bałtyk Gdynia Football Club, 81-538 Gdynia, Poland; paszul@icloud.com; 7 Faculty of Geography Tourism and Sport, University of Oradea, 410087 Oradea, Romania; pdragos@uoradea.ro

**Keywords:** muscle damage, soccer, preseason, time-motion analysis, training loads

## Abstract

The main purpose of this study is to assess the relations between training loads and selected blood parameters in professional soccer players during a preseason sports camp. Fifteen professional soccer players (age: 24.3 ± 5.25 year; height: 182.6 ± 6.75 cm; weight: 76.4 ± 6.72 kg) participated in the 12-day training camp. All the training sessions and friendly games were accurately analyzed with a GPS system. Blood samples were taken from the players and analyzed before the camp (PRE), in the middle (MID), and one day after the camp (POST). Mean total distance covered by the players during the camp was 85,205 ± 2685 m, high-intensity running 12,454 ± 1873 m, and sprinting 639 ± 219 m. The highest aspartate transaminase (AST), lactate dehydrogenase (LDH), creatine kinase (CK), and C-reactive protein (CRP) values were observed after six days of the camp. The application of intensive training during a 12-day sports camp can be associated with chronic muscle pain with high activity of some blood enzymes (CK, AST) and a high concentration of myoglobin (Mb). During training camps longer than 10 days, it would be necessary to apply, every second or third day, one day of rest, and the training load should not exceed two units every day.

## 1. Introduction

The process of sports training in soccer includes training units in the preparation, starting, and detraining (rest) periods. Periodization of this process is more widely observed in groups of typically young players, whereas in the case of professional players, the difference between the preparation and starting periods is becoming blurred and the detraining period is short, sometimes not longer than two weeks. This is due to the large number of league, cup, and friendly matches (up to 80) played by soccer players in the annual training cycle [1]. Sports camps organized by professional soccer clubs are a common form of training organization in the preparation period, during which players do classic exercising and play control matches. Sports camps last from 7 to 14 days and are organized, especially in Mediterranean countries, by soccer teams from Central and Northern Europe, where weather conditions during winter do not allow to train on natural grass pitches. Due to the accumulation of a large number of training units, including high-intensity exercises and control matches, players respond with post-exercise overload and even acute fatigue that can become chronic [2].

The most common symptom of player fatigue is skeletal muscle damage, which is shown by the increase in biochemical indicators such as: creatine kinase (CK), lactate dehydrogenase (LDH), myoglobin (Mb), aspartate transaminase (AST), and alanine transaminase (ALT). There is even degradation of purine nucleotides in muscle tissue resulting in the impairment of their functionality [3]. Internal organs, e.g., liver (ALT, AST), are also damaged. During workout overload, systemic inflammation (CRP—C-reactive protein) is also revealed. According to Birch et al. [4], repeated physical exercise without adequate rest causes eccentric muscle contraction, which results in damage to muscle structure. Large training loads used in soccer training for a long period of time, e.g., over two or three weeks, can lead to metabolic and organ overloads and, consequently, to chronic fatigue. Therefore, the quantity and quality of training loads is a basic risk factor for adverse fatigue changes in soccer players. The analysis of the training methods used in relation to the changes that they cause in the body of a soccer player can effectively help in the prevention of muscle damage.

In recent years, the global positioning system (GPS) movement analysis system has gained particular importance to assess the optimization of training loads in soccer [5,6]. Satellite navigation devices can be used to accurately describe players’ activity profiles, including their position and movement on the field [7,8]. The research that confirms the high validity and reliability of GPS devices for measuring movement in team sports encourage even more use in future research [5]. Although a GPS device is a useful tool for monitoring external loads, it does not register actual psychophysiological response [9]. Therefore, sports scientists working with soccer players and other sports should be aware of the limitations of GPS systems and use other tools enabling comprehensive evaluation of training load. The regular collecting and analyzing of data such as postexercise self-reports or various blood markers should be an integral part of the training process.

The majority of previous research on the issue of locomotion of professional soccer players during matches used video tracking systems [10,11,12]. A relatively small number of articles containing GPS data of elite soccer games [13,14] or training loads [15,16] is probably a consequence of a low availability of these devices to soccer clubs and the protection of the results obtained.

To our knowledge, so far only one publication has attempted to describe training loads during a preseason camp using GPS technology [17]. The authors of this publication focused mainly on the heart rate (HR) response and on the subjective estimation of training loads, using the rating of perceived exertion (RPE) in players who travelled to the camp crossing six time zones. However, they did not analyze training loads in relation to biochemical blood parameters.

Lago et al. [18], using a GPS system, showed that professional soccer players cover a distance of 9 to 14 km per match, depending on their position on the field. Other authors [19,20] have also found that, during one particular match, players covered a distance of 2.5 to 3.5 km of high intensity activity (speed > 15 km/h) and about 0.7 km of sprints (speed > 20 km/h).

In the available literature, we have not found any papers defining the structure of training loads in soccer calculated using a satellite system during the preparation period at sports camps, where players are subjected to a high physical load. This fact can be an effect of both organizational and financial difficulties [21].

The aim of this study was to evaluate the relationship between training loads and the selected blood parameters in professional soccer players during a 12-day sports camp. The hypothesis of the authors was that the high-load exercises during the sports camp will be associated with the increased level of analyzed blood parameters.

## 2. Methods

### 2.1. Participants

Fifteen high-level (Polish super league) professional soccer players (five defenders, five midfielders, and five strikers) (24.3 ± 5.25 year, 182.6 ± 6.75 cm, and 76.4 ± 6.72 kg) participated in the study. Usually they train eight times a week and play one control game in the preparation period. During the study, the tested subjects lived in the same place and were nourished in the same way. The diet of the players was standard but included an increased amount of vegetables, fruit and dairy products (a sports diet). The participation in training sessions of the analyzed players reached 100%. All the subjects had valid medical cards, received detailed information about the research procedures, and gave their written consent. The protocol was fully approved by the Ethical Committee of the local Medical Association (Nr KB—1/14).

### 2.2. Study Design

The study was conducted during a 12-day sports camp during the preparation period in July. The preparation plan consisted of the implementation of high-intensity training loads and was developed by the team coach without the intervention of the research team. The locomotion of players was recorded during nine training days. On the other days, strength training in the sports hall and a regeneration session were carried out during which it was not possible to use the GPS. In addition, fasting blood samples were taken from the players three times: in the first (PRE) and the seventh day (IN THE MIDDLE) of the camp, and on the first day after the camp (POST), to determine selected biochemical indicators as a result of training loads. The total distance covered by the soccer players during each training unit was calculated taking into account five intensity zones. The sports camp took place in Poland, in summer, at an air temperature of 22 °C to 24 °C, humidity of 55%–60%, and atmospheric pressure of 1010–1025 hPa (Table 1).

### 2.3. Time-Motion Analyses

The distance covered by the players during all training sessions was measured using previously validated [22,23] portable GPS devices (minimaxX version 4.0, Catapult Innovations, Melbourne, Australia) with 10 Hz frequency and analyzed using appropriate software (Catapult Sprint 5.0, Catapult Innovations, 2010). During the training sessions, the players wore vests with GPS units placed on the upper back. As recommended, the GPS devices were activated 15 min before the start of the training session.

The distances covered during sprinting (>7 m·s^−1^), high intensity running (HIR; 5.5–7 m·s^−1^), low intensity running (LIR; 4–5.5 m·s^−1^), jogging (2–4 m·s^−1^), and standing and walking (0–2 m·s^−1^), as well as the total distance, were measured. These speed zones were previously described by Rampinini et al. [24].

### 2.4. Blood Analyses

Blood samples were corrected for hemoconcentration and analyzed in accordance with the indexes of muscle damage of Dill and Costill [25]. The reference values for the tested variables were: alanine transaminase ALT (<38.0 U/L), aspartate aminotransferate AST (<40 U/L), lactate dehydrogenase LDH (80–285 U/L), C-reactive protein CRP (<5.0 mg/L), creatine kinase CK (<160 U/L), and Myoglobin Mb (10–46 ng/mL) [26]. Blood samples were collected from the ulnar vein in the morning of the first and the seventh day of the analyzed period and one day (morning) after the sports camp. Biochemical analyses were conducted using an A-15 analyzer (Biosystems SA, Costa-Brava, Barcelona, Spain, 2012).

### 2.5. Statistical Analyses

The results are presented as mean and standard deviation (M ± SD). All of the data sets were assessed using the Shapiro–Wilk test to check normality. Repeated-measures ANOVA with a post-hoc Tukey test were applied in homogeneous variables. For heterogeneous results, Friedman’s ANOVA with appropriate post hoc test was applied. The intraclass correlation coefficient—ICC_(1)_ was calculated to determine the reliability of physiological variables measurements according to Bartko [27] and Liljequist et al. [28]. The ICC_(1)_ values were interpreted as “poor” (ICC < 0.5), “moderate” (0.5–0.75), “good” (0.75–0.9), and “excellent” (ICC > 0.9) [27]. The level of significance was set at *p* < 0.05. All statistical analyses were conducted using StatSoft Statistica software version 9.0 (Statsoft, Tulsa, OK, USA).

## 3. Results

During the nine training days in which the locomotion was measured, the longest distance was covered by the players in the low intensity zone (jogging and low intensity running), while the shortest distance was that of sprints. On average, the players ran on each training 5680.3 m (Table 2).

**Figure 1:**
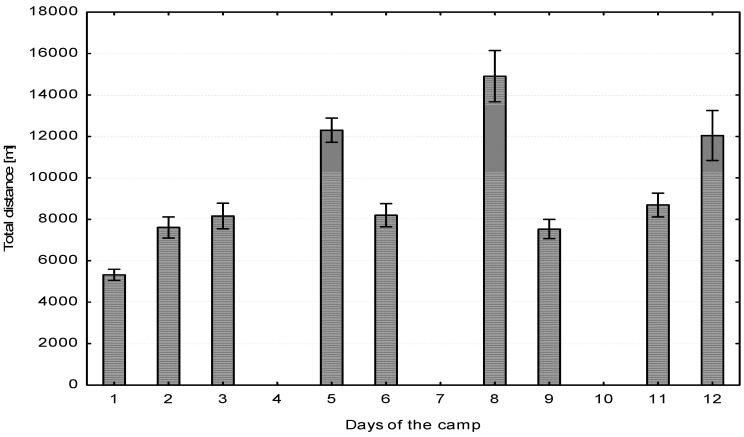
Total distance covered during each day of the camp.

**Figure 2:**
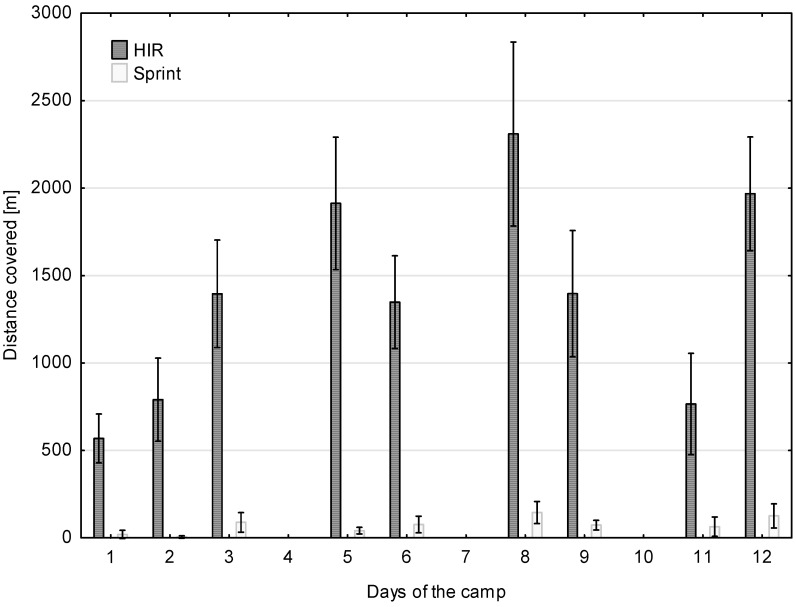
Distance covered in high intensity running (HIR) and sprinting during each day of the camp.

Taking into account the components of the total distance, it was found that the players were most active in low intensity (4–5.5 m·s^−1^) while performing training tasks that enhance techniques and tactics (Table 1). The plan of training loads assumed a gradual increase in the running activity of the players in the first three days of the camp and then, on the fourth day, strength training was implemented. This applies to the total distance covered by the players as well as to high-intensity running (HIR). On the fourth day, a post-training regeneration session and strength training in the sports hall were implemented with the players. In the following days (5 to 9), a similar cycle was implemented, i.e., two days of high-load training (total distance between 12,000 and 14,000 m) and high-intensity running (HIR about 2000 m) interrupted by one day of wellness, fitness, and general strength exercises. In the last two days of the training camp, the training load was reduced and the training intensity was increased (Figure 1 and Figure 2).

In Figure 2 it can be seen that the lowest number of sprints was implemented in the first two days of the camp, while most of the sprints were made in the third, eighth and twelfth, at equal time intervals.

The level of biochemical blood indicators in players varied in the different days of the sports camp. Moderate reliability was calculated for CRP and Mb. ICC_(1)_ values for AST, ALT, and CK were defined as “good”, and the highest reproducibility was found for LDH. The highest AST, LDH, CK, and CRP values were obtained after six training days (in the middle of the camp duration) and the highest myoglobin value was obtained in the final phase of the camp. The differences in average values of the measured indicators in the three phases of the sports camp were statistically significant in terms of AST, CK, CRP, and myoglobin (Table 3).

## 4. Discussion

There are not many studies regarding the association between training loads and the level of fatigue of professional football players at sports camps [17,29,30]. The problem particularly applies to those players who have been on sports training camps for over 10 days and are subjected to high training loads. A high level of blood indicators such as CK, Mb, AST, and ALT has been found in this study. This is probably the result of applying explosive strength exercises, high-intensity runs, and low-intensity but high-volume exercises to the players [31,32]. However, obtained CRP values indicate that generalized inflammation in the players’ bodies has not been found (Table 3).

Halson and Jeukentrup [33] found that high training loads cause a high risk of fatigue and overtraining of the players’ bodies. This is possible at every stage of training when applying long-term irrational training loads. During sports camps, an accumulation of high-intensity exercises occurs in a relatively short time and short recovery periods after effort may not compensate fatigue changes.

This research study was conducted on professional soccer players of the highest soccer league in Poland—the Ekstraklasa. The most usual form of preparation of players of this level at the beginning of the preparation period is a sports camp lasting from 6 to even 14 days. Taking into consideration the possibility of high fatigue effects in players, the training plan was designed so that players could have regeneration days, characterized by reduced volume and intensity of training (Table 1).

The biggest muscle fatigue during the soccer training process occurs at the beginning of the preparation period, after a rest break of two or three weeks (detraining period). Therefore, sports scientists and coaches should not implement training loads with too much volume and intensity of exercise at the beginning of a sports camp. Acute fatigue effects can be observed in players after only three days of training (i.e., six training units). In our research, the training program included reduced volume and training intensity after the third day and then every other day of the sports camp. Despite this, after six days a statistically significant increase in CK and Mb was observed in the players. Due to the continuous control of biochemical indicators and the distance covered by the players, the positive response of the training staff to fatigue changes was the reduction of training loads (volume—Figure 1 and intensity—Figure 2) on days 9, 10, and 11. This practice is commonly used by sport scientists working in elite soccer [17,34,35]. After obtaining the increased values of CK, AST, and LDH in the middle of the camp, the training load was adapted. It is widely known that eccentric exercises induce muscle damage followed by CK leakage [36]. Therefore, the number of exercises including eccentric work and running at high intensities was reduced. This action was probably associated with the lower values of analyzed blood parameters at the end of the camp.

The significant increase in Mb during this time can be explained by the reaction of the players’ bodies to relatively large training loads during the entire 12-day sports camp.

There are a few papers concerning the analysis of biochemical blood indicators in soccer players during a sports camp. Buchheit et al. [29] studied changes in CK concentration in blood serum of professional soccer players during an 11-day sports camp. Similar to our study, these authors found significant changes in the activity of CK in the blood serum of players in the different days of the sports camp. Some papers discuss the changes in blood indicators that characterize skeletal muscle fatigue in soccer players, with different levels of training after a match, after a training period, or after a single workout with a certain load. At the end of the preparation period, Alves et al. [37] reported CK at 626 U/L 48 h after the match and the variation of this indicator among players was high (from 141 to 1830 µ/L). Similar values of this indicator were observed by Mohr et al. [1] 24 h after three matches played in one week: 1043 ± 76, 1252 ± 130 and 1011 ± 114 U/L. In addition, similar values of this indicator (900 U/L) were reported by Fatouos et al. [38] after a soccer match. Soccer is one of the few sports in which players have CK values above the normal range after the match, and this may be related to skeletal muscle damage. High values of AST, ALT, and LDH were also confirmed in our study (Table 3). Gomes et al. [39] also observed similar correlations in tennis players after simulated matches. These authors observed CK values from 105 to 498 U/L 24 h after the match and the return to reference values lasted at least 48 h. According to these authors, the effect of muscle fatigue, after a match or repetitive training units during a sports camp, may be caused by inadequate regeneration of the players’ bodies as a result of rest breaks that are too short. Generalized inflammations in the players’ bodies can be an evidence of the incorrect use of high-intensity training loads in a short time (during sports camps). The CRP indicator may be their symptom. In our study, high CRP values were not observed in soccer players, i.e., above the reference range. Mohr et al. [1] obtained similar results in the soccer players before (0.9 ± 0.1 mg/L) and after (1.3 ± 0.0 mg/L) the preparation period. Similar conclusions were presented by Siahkouhian and Esmaeilzadeh [40], who examined young soccer players in long-term training and found that the players had a stable CRP level (0.33 ± 0.13 mg/dL).

Mb concentration in the blood serum of players is an important factor in the assessment of aerobic endurance in soccer training. A high level of Mb with high CK activity after physical effort, especially of a mixed-intensity (aerobic–anaerobic), may be connected with the eventual appearance of delayed-onset muscle soreness (DOMS).

Gomes et al. [39] found high CK and Mb (from 81 to 175 ng/mL) examining young tennis players after a simulated 3-h match. They stated that the return of Mb and CK to reference values lasted 48 h. Fransson et al. [41] examined the effects of fatigue in various muscle groups in soccer players after a simulated soccer game. The authors stated that CK activity was the highest 24 h after the match and did not reach reference values 48 h after exercise. Moreover, Mb in blood serum was the highest immediately after the match. After 24 h rest, it was at a level close to the reference values and after 48 h it reached its resting level. Therefore, applying three days of training loads with reduced volume and intensity of effort to soccer players can have a positive effect avoiding acute muscle fatigue.

Some limitations of the current study warrant mention. High values of standard deviations for some variables (e.g., CK) makes the relevance of statistical significance questionable. Previous research shows that CK activity varies individually according to such factors as percentage of fast twitch muscle fibres or race [42]. The number of participants in our study was relatively low (n = 15) and included players from different backgrounds. For example, the highest values of CK were obtained by an African player. Therefore, large SD values seem to be justified. However, future research should be accomplished involving a bigger sample to improve the reliability of statistical analysis. Moreover, the inability to provide the data about potential relationships between blood parameters and mechanical indicators of fatigue is another limitation. Unfortunately, there was no agreement from the coaching staff for any additional mechanical tests at this stage of preseason.

## 5. Conclusions

Sports camps in soccer training are an important element in preparing players for league games. Based on our research, we found that the application of training loads during a 12-day sports camp can be associated with a high activity of some blood enzymes (CK, AST, ALT) and a high concentration of Mb.

According to the findings of our study, soccer camps for professional players should not last longer than 7 days, with one day of rest or lower intensity and volume of training loads. In case of sports camps lasting more than 10 days, it would be necessary to apply, every second or third day, one day of rest, and the training load should not exceed two units every day.

## Figures and Tables

**Table 1:** Overview of the 12-day training load during the sports camp in the preparation period.

Days of Sports Camp		Training Drills	
		Morning	Afternoon
1.	Monday	Speed, technique, tactics	Core, coordination
2.	Tuesday	Explosive power, coordination	Speed, small-sided games
3.	Wednesday	Stretching, technique	Tactics, control game
4.	Thursday	Regeneration	Fitness, strength
5.	Friday	Plyometrics, small-sided games	Speed, coordination, technique
6.	Saturday	Technique, tactics	Tactics, control game
7.	Sunday	Regeneration, recreation	Fitness, strength
8.	Monday	Interval run, technique	Core, tactics
9.	Tuesday	Strength, plyometrics, coordination	Speed, small-sided games
10.	Wednesday	Regeneration	Fitness, strength
11.	Thursday	Speed, technique	Coordination, tactics
12.	Friday	Stretching, technique	Control game

**Table 2:** Distance covered by the players in each of the speed zones during the training camp.

Speed Zones	Standing and Walking [m]	Jogging [m]	LIR [m]	HIR [m]	Sprint [m]	Total Distance [m]
Mean	11,315.0	26,996.3	33,800.9	12,453.5	639.3	85,205.0
SD	1644.5	1466.7	3321.9	1873.3	218.6	2684.6
% of total distance	13.28	31.68	39.67	14.62	0.75	100

LIR—low-intensity running, HIR—high-intensity running, SD—standard deviation.

**Table 3:** Changes in blood biochemical indicators in the three phases of a 12-day sports camp in professional soccer players.

INDICATOR	1 PRE	2 IN THE MIDDLE	3 POST	ICC_(1)_
AST [U/L]	40.67 ± 15.97	45.07 ± 16.98^2 ÷ 3^ *	35.73 ± 9.97	0.80
ALT [U/L]	45.87 ± 15.02	44.00 ± 14.06	38.93 ± 9.56	0.88
CK [U/L]	569.53 ± 337.89^1 ÷ 2^ *	692.93 ± 371.17^2 ÷ 3^ *	453.33 ± 279.40	0.87
LDH [U/L]	261.67 ± 48.56	273.73 ± 53.67	268.67 ± 59.65	0.91
CRP [mg/L]	1.44 ± 0.77^1 ÷ 3^ *	1.16 ± 0.46	0.83 ± 0.34	0.67
Myoglobin [ng/mL]	23.93 ± 8.75^1 ÷ 3^ *	25.80 ± 9.52^2 ÷ 3^ *	34.13 ± 8.03	0.75

* Significant differences at *p* < 0.05; AST—aspartate transaminase, ALT—alanine transaminase, CK—creatine kinase, LDH—lactate dehydrogenase, CRP—C-reactive protein, ICC_(1)_—intraclass correlation coefficient.
